# A Classical Genetic Solution to Enhance the Biosynthesis of Anticancer Phytochemicals in *Andrographis paniculata* Nees

**DOI:** 10.1371/journal.pone.0087034

**Published:** 2014-02-25

**Authors:** Alireza Valdiani, Daryush Talei, Soon Guan Tan, Mihdzar Abdul Kadir, Mahmood Maziah, Mohd Yusop Rafii, Sreenivasa Rao Sagineedu

**Affiliations:** 1 Department of Biochemistry, Faculty of Biotechnology and Biomolecular Sciences, Universiti Putra Malaysia, Serdang, Selangor DE, Malaysia; 2 Department of Cell and Molecular Biology, Faculty of Biotechnology and Biomolecular Sciences, Universiti Putra Malaysia, Serdang, Selangor DE, Malaysia; 3 Medicinal Plant Research Centre, Shahed University, Tehran, Iran; 4 Department of Agriculture Technology, Faculty of Agriculture, Universiti Putra Malaysia, Serdang, Selangor DE, Malaysia; 5 Institute of Tropical Agriculture, Universiti Putra Malaysia, Serdang, Selangor DE, Malaysia; 6 Department of Pharmaceutical Chemistry, School of Pharmacy, International Medical University, Kuala Lumpur, Malaysia; Cairo University, Egypt

## Abstract

Andrographolides, the diterpene lactones, are major bioactive phytochemicals which could be found in different parts of the medicinal herb *Andrographis paniculata*. A number of such compounds namely andrographolide (AG), neoandrographolide (NAG), and 14-deoxy-11,12-didehydroandrographolide (DDAG) have already attracted a great deal of attention due to their potential therapeutic effects in hard-to-treat diseases such as cancers and HIV. Recently, they have also been considered as substrates for the discovery of novel pharmaceutical compounds. Nevertheless, there is still a huge gap in knowledge on the genetic pattern of the biosynthesis of these bioactive compounds. Hence, the present study aimed to investigate the genetic mechanisms controlling the biosynthesis of these phytochemicals using a diallel analysis. The high performance liquid chromatography analysis of the three andrographolides in 210 F_1_ progenies confirmed that the biosynthesis of these andrographolides was considerably increased via intraspecific hybridization. The results revealed high, moderate and low heterosis for DDAG, AG and NAG, respectively. Furthermore, the preponderance of non-additive gene actions was affirmed in the enhancement of the three andrographolides contents. The consequence of this type of gene action was the occurrence of high broad-sense and low narrow-sense heritabilities for the above mentioned andrographolides. The prevalence of non-additive gene action suggests the suitability of heterosis breeding and hybrid seed production as a preferred option to produce new plant varieties with higher andrographolide contents using the wild accessions of *A. paniculata*. Moreover, from an evolutionary point of view, the occurrence of population bottlenecks in the Malaysian accessions of *A. paniculata* was unveiled by observing a low level of additive genetic variance (*V_A_*) for all the andrographolides.

## Introduction

**Figure 1:**
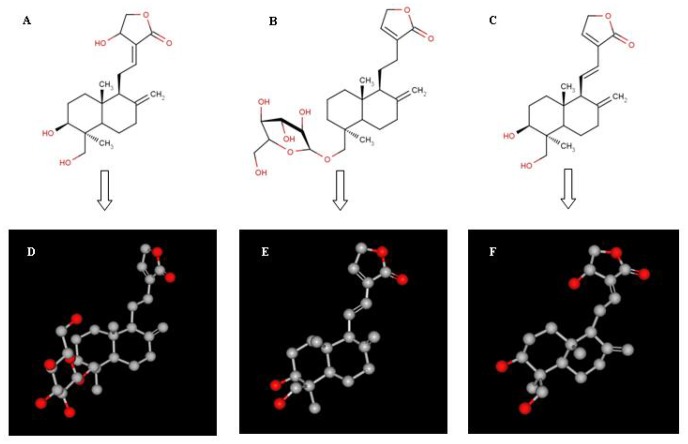
A, B and C refer to two-dimensional forms of AG, NAG and DDAG, while, D, E and F refer to three-dimensional structures of AG, NAG and DDAG, respectively. R: Glucose.

*Andrographis paniculata* (hereafter AP) is a well-known traditional medicinal plant species with a bright economic horizon belonging to the Acanthaceae family [1]. The presence of many bioactive constituents from different chemical compound classes such as flavonoids, diterpene lactones (in free and glycosidic forms), phenylpropanoids and xanthones [2], [3] has been confirmed in AP. Many therapeutic properties of AP and its bioactive principles have been reviewed extensively [1]. Among these constituents, three principle diterpenoid-based compounds including andrographolide (AG) [4], [5], neoandrographolide (NAG) [6] and 14-deoxy-11,12-didehydroandrographolide (DDAG) [7], shown as Figure 1A–F, have received more attention because of their potential therapeutic effects in hard-to-treat diseases such as cancer [8], HIV [9], hepatitis [10] and diabetes [11]. This in turn has led to a rising price and market demand for AP-derived products. Quality dry leaves of AP are sold for as much as US$5/kg, whilst the purified andrographolide and its derivatives could reach up to US$100,000/kg [12]. The latest pricing by Sigma-Aldrich Corporation (USA) in 2013 for the 100 and 500 mg packages of andrographolide 98% is US$36.20 and US$135.00, respectively.

Taking these into account, the investigation of the potential approaches which could possibly lead to an increase in the production of the three andrographolides becomes an attractive issue. In light of this, the impacts of different factors such as the plant growth regulators (PGRs) [13], [14], enzymes [15] light intensity [16], integrated nutrient systems [17] spacing and plant density [18], [19] on increasing the andrographolides contents in AP have been recently studied. Jebril et al. [20] and Rajpar et al. [21] surveyed the accumulation of andrographolides in Malaysian AP accessions under normal and saline soils, separately. Reportedly, the ranges of AG, NAG and DDAG were between 0.25–1.00% vs. 2.6–3.9%, 0.11–0.26% vs. 1.4–2.1%, and 0.12–0.31% vs. 0.19–0.27%, in normal and saline conditions, respectively [20], [21]. Herein, we have strived to ascertain whether the mentioned rates are genetically increasable or not and to achieve that, a classic approach namely diallel cross was employed. The term diallel is a Greek word first used by Schmidt [22] and implies all possible crosses among a collection of male and female individuals [24]. In fact, a diallel cross is a mating scheme to examine the genetic underpinning of quantitative traits [25]. Prior to the diallel cross, experiments were conducted to obtain the intraspecific hybridization technology through finding the best time to carry out the cross pollinations using some morphological (stigmatic) and phenological indices [23]. To the best of our knowledge, the present research was the first attempt to implement the diallel mating design on AP (Table 1) to assess the biosynthesis of AG, NAG and DDAG, and finally to analyze the genetic basis of these three anticancer phytochemicals in this plant. The acquired findings could offer an enormous potential to develop new varieties with a higher content of the phytochemicals.

**Table 1:** Hybridization scheme of the seven parental AP accessions.

Code	Hybrid	Pistillate ♀	Staminate ♂
P1	P1×P1	11179SE	11179SE
P2	P2×P2	11216NS	11216NS
P3	P3×P3	11261PE	11261PE
P4	P4×P4	11313PA	11313PA
P5	P5×P5	11322PA	11322PA
P6	P6×P6	11344KE	11344KE
P7	P7×P7	11350TE	11350TE
H1	P1×P2	11179SE	11216NS
H2	P1×P3	11179SE	11261PE
H3	P1×P4	11179SE	11313PA
H4	P1×P5	11179SE	11322PA
H5	P1×P6	11179SE	11344KE
H6	P1×P7	11179SE	11350TE
H7	P2×P3	11216NS	11261PE
H8	P2×P4	11216NS	11313PA
H9	P2×P5	11216NS	11322PA
H10	P2×P6	11216NS	11344KE
H11	P2×P7	11216NS	11350TE
H12	P3×P4	11261PE	11313PA
H13	P3×P5	11261PE	11322PA
H14	P3×P6	11261PE	11344KE
H15	P3×P7	11261PE	11350TE
H16	P4×P5	11313PA	11322PA
H17	P4×P6	11313PA	11344KE
H18	P4×P7	11313PA	11350TE
H19	P5×P6	11322PA	11344KE
H20	P5×P7	11322PA	11350TE
H21	P6×P7	11344KE	11350TE

P: parental plant, H: hybrid, SE: Selangor, NS: Negeri Sembilan, PE: Perak, PA: Pahang, KE: Kelantan, TE: Terengganu, ♀: female parent, ♂: male parent.

## Results

### High Performance Liquid Chromatography (HPLC) Method Efficiency

**Figure 2:**
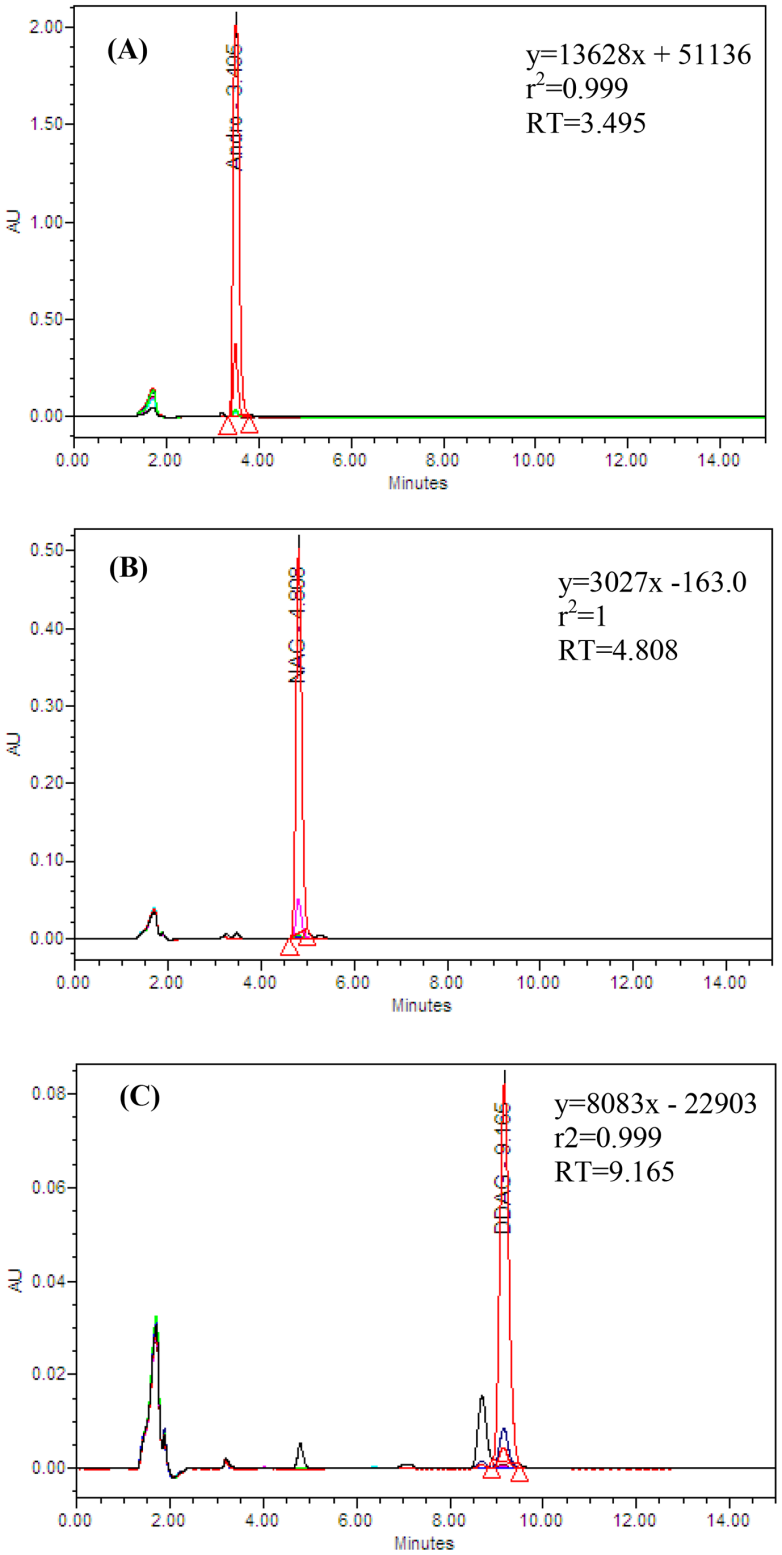
HPLC chromatogram of (A) AG, (B) NAG and (C) DDAG.

The retention times (RT) and the coefficient of determinations (r^2^ = 0.999–1) of andrographolide (AG), neoandrographolide (NAG) and 14-deoxy-11,12-didehydroandrographolide (DDAG) confirmed the efficiency of the method (Fig. 2A–C), and LODs for AG, NAG and DDAG were 0.30, 0.18 and 0.26 µg/mL, respectively. Likewise, the measured LOQs for AG, NAG and DDAG were 1.0, 0.96 and 0.91 µg/mL, respectively. Apart from the main results, as a technical point, the efficacy of the isocratic method was verified by a high coefficient of determination for the compounds. Besides, the decreased retention times of the three components led to saving chemicals and time as well as reducing the depreciation of HPLC instrument.

### Analysis of Variance (ANOVA)

The outlines of the diallel ANOVA are presented in Table 2. As mentioned earlier, the field pot trial was undertaken as an efficient alternative strategy for normal field trial to reduce the experimental errors and environmental effects, thereby increasing the precision and replicability of the experimental findings. The analysis of variance (ANOVA) results revealed that the technique was accurate enough as no significant difference was observed among replicates except for andrographolide percentage (AGP), while the relatively low coefficient of variation (C.V) of the traits confirmed the reliability of the method (Table 3). Interestingly, the first clue of heterotic behavior appeared in the ANOVA results in which the 28 genotypes including the 7 parents and 21 hybrids were significantly different (*P*≤0.01) in all the traits (Table 3). The ANOVA revealed a greater mean square of specific combining ability (SCA) than general combining ability (GCA) for AG and DDAG components. This complied with the greater importance of non-additive gene effects than the additive gene effects for these two phytochemicals. A converse trend happened to the neoandrographolide components (NAGP, NAGC and NAGY), indicating the predominance of additive gene action over non-additive gene effects in the inheritance of NAG (Table 3).

**Table 2:** The ANOVA outlines for a half diallel analysis in Griffing’s method 2.

Source of variation	d.f.	Expected meansquares
Replications	(r−1)	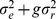
Genotypes	{[n(n−1)/2]+n}−1	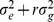
GCA	n−1	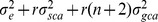
SCA	n(n−1)/2	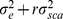
Error	(r−1) {[n(n−1)/2]+n}−1	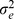

r and n refer to the number of replications and parents, respectively [31], [41]._
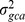
_: the variance of GCA,

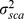
: the variance of SCA, 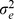: the variance of error, 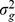: the variance of genotype, 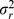: the variance of replication, g: genotype.

**Table 3:** ANOVA for combining ability of the three phytochemicals in AP.

S.O.V	d.f.	Mean squares								
		AGP	AGC	AGY	NAGP	NAGC	NAGY	DDAGP	DDAGC	DDAGY
Replication	9	0.29*	0.02^ns^	1095.08^ns^	0.00^ns^	0.00^ns^	1.43^ns^	0.06^ns^	0.006^ns^	292.63^ns^
Genotype	27	0.97**	0.14**	6905.26**	0.003**	0.001**	77.40**	2.07**	0.20**	10125.02**
GCA	6	0.78**	0.042**	2090.97**	0.006**	0.002**	110.30**	0.30^ns^	0.03^ns^	1873.77^ns^
SCA	21	1.03**	0.16**	8280.78**	0.003**	0.001**	68.00**	2.57**	0.25**	12482.52**
Error	243	0.14	0.01	681.60	8.51	3.76	1.31	0.03	0.004	197.64
C.V (%)		19.86	20.93	20.92	6.77	14.99	12.63	17.64	19.26	19.17

ns: non-significant, **, *significant at *P*≤0.01 and *P*≤0.05 respectively. S.O.V: source of variation, d.f: degree of freedom, AGP: AG percentage per plant (%), AGC: AG content per plant (g), AGY: AG yield per hectare (kg/ha), NAGP: NAG percentage per plant (%), NAGC: NAG content per plant (g), NAGY: NAG yield per hectare (kg/ha), DDAGP: DDAG percentage per plant (%), DDAGC: DDAG content per plant (g), DDAGY: DDAG yield per hectare (kg/ha).

### Anticancer Phytochemicals in the Hybrids and Parents

**Figure 3:**
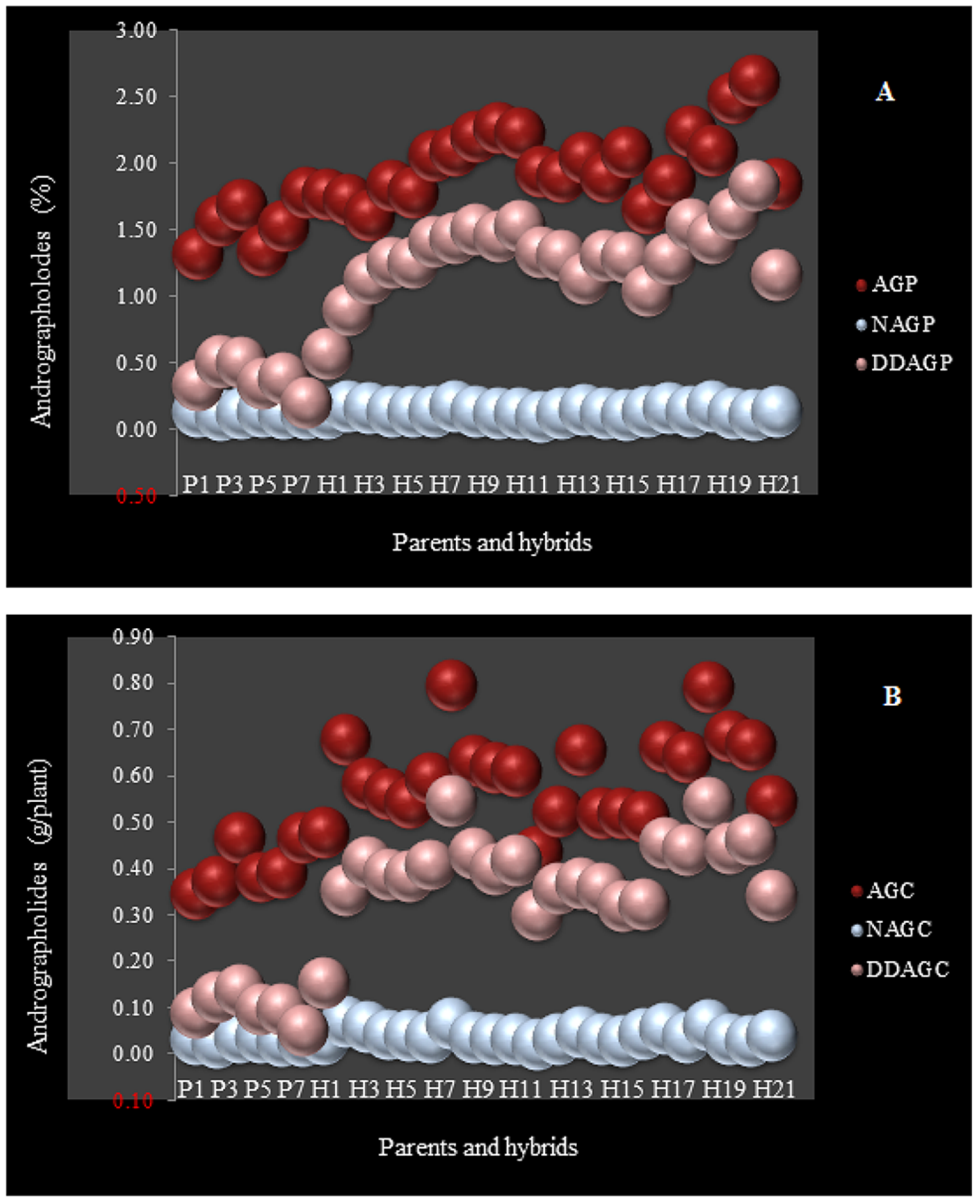
Distribution of AG, NAG and DDAG among the 28 parental and hybrid AP plants. Each sphere represents one plant from left to right in the order of the seven parents (P1–P7) to the 21 hybrid (H1–H21) in the x-axis. The numbers in the y-axis represent (A) percentages of the three phytochemicals in the basis of dry weight per plant, and (B) the dry weight-based contents of the same phytochemicals in g/plant. AGP: AG percentage per plant (%), AGC: AG content per plant (g), NAGP: NAG percentage per plant (%), NAGC: NAG content per plant (g), DDAGP: DDAG percentage per plant (%), DDAGC: DDAG content per plant (g).

Heterosis was evidenced again by Duncan’s multiple comparison test at *P≤*0.01 and a significant difference between the parental plants and hybrids was confirmed (Table 4). Figure 3 is a graphical presentation of the percentages and the contents of the three phytochemicals. An obvious boost was detected in the hybrids compared with their parents. However, in practice, the contents of the three anticancer agents were more applicable, because in addition to the percentages of the phytochemical, the dry yield of each plant was reflected in it. Accordingly, the highest andrographolide content (AGC), neoandrographolide content (NAGC) and 14-deoxy-11,12-didehydroandrographolide content (DDAGC) all belonged to the hybrids H6 and H18 with yields of 0.79, 0.06 and 0.55 g/plant, respectively. In addition, P7 and P3 were the best parental accessions according to their higher AGC, NAGC and DDAGC (Fig. 3 and Table 4). The parental accessions P1, P2 and P6 had the lowest AGC, NAGC and DDAGC, respectively. The hybrids H10 and H14 produced the lowest amount of the three phytochemicals, but the reduction of AGC in hybrid H10 was more critical, whereas it dramatically decreased less than some of the parental individuals such as P6 and P7. The NAGC level dropped drastically down to 0.02 g/plant in both H10 and H14 hybrids, which was even lower than all the parental plants except P2 (Fig. 3 and Table 4). Hybrid H6 (P1×P7) produced the highest yields of andrographolide (AGY), neoandrographolide (NAGY) and 14-deoxy-11,12-didehydroandrographolide (DDAGY) with 177.2, 14.8 and 121.7 kg/ha, respectively (Table 4).

**Table 4:** Means comparison test of the three phytochemicals in the 28 parental and hybrid AP plants.

Plants	Means								
	AGP	AGC	AGY	NAGP	NAGC	NAGY	DDAGP	DDAGC	DDAGY
P1	1.32^a^	0.34^a^	77.1^a^	0.13^efgh^	0.03^def^	7.9^def^	0.33^ab^	0.09^ab^	19.8^ab^
P2	1.57^bc^	0.37^ab^	83.4^ab^	0.11^b^	0.02^ab^	6.1^ab^	0.51^bc^	0.12^bc^	27.3^bc^
P3	1.69^abcd^	0.46^bcd^	104.2^bcd^	0.13^cdef^	0.03^def^	8.1^def^	0.50^bc^	0.14^bc^	30.8^bc^
P4	1.35^a^	0.38^ab^	85.2^ab^	0.13^efghi^	0.03^efg^	8.4^efg^	0.34^ab^	0.09^abc^	21.6^abc^
P5	1.53^ab^	0.39^ab^	87.1^ab^	0.12^bcd^	0.03^bcd^	7.0^bcd^	0.38^ab^	0.09^abc^	21.9^abc^
P6	1.79^bcd^	0.46^bcd^	103.7^bcd^	0.12^bcd^	0.03^bcd^	7.1^bcd^	0.20^a^	0.05^a^	11.8^a^
P7	1.77^bcd^	0.47^bcde^	106.4^bcde^	0.12^cde^	0.03^cde^	7.7^cde^	0.57^c^	0.15^c^	34.5^c^
H1	1.74^bcd^	0.67^j^	150.9^j^	0.18^m^	0.07^m^	15.9^m^	0.90^d^	0.35^defg^	78.9^defg^
H2	1.62^ab^	0.58^defghij^	129.9^defghij^	0.16^kl^	0.06^k^	13.5^k^	1.13^ef^	0.41^ghi^	91.6^ghi^
H3	1.83^bcde^	0.56^defghij^	125.1^defghij^	0.14^hij^	0.04^hij^	9.6^hij^	1.26^fg^	0.38^efgh^	86.2^efgh^
H4	1.79^bcd^	0.54^cdefghi^	121.1^defghi^	0.14^ghij^	0.04^ghi^	9.5^ghi^	1.26^fg^	0.38^efgh^	85.4^efgh^
H5	2.06^cdefg^	0.59^efghij^	132.7^efghij^	0.13^efgh^	0.03^efgh^	8.5^efgh^	1.41^ghij^	0.41^ghi^	91.2^ghi^
H6	2.09^defg^	0.79^k^	177.2^k^	0.17^lm^	0.06^l^	14.8^l^	1.44^ghijk^	0.55^j^	121.7^j^
H7	2.20^efgh^	0.63^fghij^	141.0^fghi^j	0.13^efgh^	0.03^efg^	8.5^efg^	1.50^hijk^	0.43^hi^	96.5^hi^
H8	2.27^gh^	0.61^fghij^	137.4^fghij^	0.12^cde^	0.03^cde^	7.7^cde^	1.45^ghijk^	0.39^fghi^	88.7^fghi^
H9	2.22^efgh^	0.61^fghij^	135.7^fghij^	0.12^cde^	0.03^cde^	7.7^cde^	1.53^ijk^	0.42^ghi^	93.2^ghi^
H10	1.94^bcdefg^	0.43^abc^	97.5^abc^	0.10^a^	0.02^a^	5.2^a^	1.33^fghi^	0.30^d^	67.3^d^
H11	1.91^bcdefg^	0.52^cdefg^	116.4^cdefg^	0.12^cde^	0.03^cde^	7.7^cde^	1.31^fgh^	0.36^defg^	80.1^defg^
H12	2.05^cdefg^	0.65^hij^	146.3^hij^	0.14^j^	0.04^j^	10.7^j^	1.14^ef^	0.36^efg^	81.8^efg^
H13	1.94^bcdefg^	0.53^cdefgh^	119.2^cdefgh^	0.12^cde^	0.03^def^	7.8^def^	1.33^fghi^	0.37^efg^	82.0^efg^
H14	2.09^defg^	0.52^cdefg^	115.6^cdefg^	0.11^b^	0.02^b^	6.4^b^	1.29^fg^	0.32^de^	71.7^de^
H15	1.66^abc^	0.51^cdef^	114.2^cdef^	0.14^ij^	0.04^ij^	9.7^ij^	1.05^de^	0.33^de^	72.2^de^
H16	1.88^bcdefg^	0.66^ij^	147.7^ij^	0.16^k^	0.05^k^	12.9^k^	1.29^fg^	0.45^i^	101.3^i^
H17	2.24^fgh^	0.64^ghij^	142.5^ghij^	0.13^defg^	0.03^efg^	8.5^efg^	1.54^jk^	0.44^hi^	97.8^hi^
H18	2.09^defg^	0.79^k^	176.2^k^	0.17^lm^	0.06^l^	14.7^l^	1.44^ghijk^	0.54^j^	121.1^j^
H19	2.50h^i^	0.68^j^	152.6^j^	0.12^cde^	0.03^cde^	7.6^cde^	1.63^k^	0.44^hi^	99.1^h^
H20	2.63^i^	0.66^ij^	148.6^ij^	0.11^b^	0.02^bc^	6.6^bc^	1.83^l^	0.46^i^	103.2^i^
H21	1.85^bcdef^	0.55^cdefghi^	121.7^cdefghi^	0.13^fghi^	0.04^fghi^	8.9^fghi^	1.16^ef^	0.34^def^	76.4^def^

Different letters indicate significant difference among accessions using Duncan’s multiple comparison test at *P<0.01*. AGP: AG percentage per plant (%), AGC: AG content per plant (g), AGY: AG yield per hectare (kg/ha), NAGP: NAG percentage per plant (%), NAGC: NAG content per plant (g), NAGY: NAG yield per hectare (kg/ha), DDAGP: DDAG percentage per plant (%), DDAGC: DDAG content per plant (g), DDAGY: DDAG yield per hectare (kg/ha).

### General Combining Ability (GCA)

The estimates of GCA effects of the traits are presented in Table 5. These results exhibited that the estimates of GCA effects of the phytochemical characteristics significantly varied among the accessions. Nonetheless, parent P7 consistently showed a positive and highly significant GCA estimates for AG and DDAG (0.03** and 0.03**), whilst parent P1 had the similar role for NAGC (0.01**) as shown in Table 5. Therefore, parent P7 was generally the best combiner in terms of AG and DDAG contents, and parent P1 was an excellent combiner for NAG content compared to the other accessions (Table 5).

**Table 5:** GCA and SCA estimates of the three phytochemicals in the seven parental AP accessions.

Parents/Hybrids	GCA estimates								
	AGP	AGC	AGY	NAGP	NAGC	NAGY	DDAG	DDAGC	DDAGY
P1	−0.17**	0.00^ns^	−0.79^ns^	0.01**	0.01**	1.65**	−0.0**	0.00^ns^	0.90^ns^
P2	0.01^ns^	−0.03*	−5.82*	−0.01**	0.00**	−0.88**	0.02^ns^	−0.01*	−3.0*
P3	−0.05^ns^	−0.01^ns^	−2.96^ns^	0.00^ns^	0.00^ns^	−0.01^ns^	−0.0**	−0.02**	−3.4**
P4	−0.03^ns^	0.02*	5.26*	0.01**	0.00**	0.90**	−0.0^ns^	0.02**	3.72**
P5	0.08*	0.00^ns^	−0.23^ns^	−0.01**	0.00**	−0.74**	0.08**	0.01^ns^	2.14^ns^
P6	0.11**	−0.01^ns^	−3.12^ns^	−0.01**	−0.01**	−1.48**	−0.0^ns^	−0.03**	−6.6**
P7	0.05^ns^	0.03**	7.66**	0.00**	0.00**	0.56**	0.06**	0.03**	6.37**
	**SCA estimates**								
H1	−0.02^ns^	0.1**	32.79**	0.04**	0.03**	6.00**	−0.14**	0.03^ns^	7.76*
H2	−0.08^ns^	0.04^ns^	8.87^ns^	0.02**	0.01**	2.79**	0.17**	0.09**	20.9**
H3	0.12^ns^	−0.02^ns^	−4.17^ns^	−0.01**	−0.0**	−2.0**	0.25**	0.04*	8.21*
H4	−0.03^ns^	−0.01^ns^	−2.64^ns^	0.00^ns^	0.00^ns^	−0.5^ns^	0.15**	0.04*	9.01*
H5	0.21*	0.05^ns^	11.82^ns^	−0.01*	0.00^ns^	−0.7*	0.40**	0.11**	23.5**
H6	0.30**	0.2**	45.55**	0.02**	0.02**	3.49**	0.36**	0.18**	41.1**
H7	0.32**	0.1**	25.01**	0.00^ns^	0.00^ns^	0.27^ns^	0.42**	0.13**	29.6**
H8	0.37**	0.06^ns^	13.21^ns^	−0.01**	−0.0**	−1.4**	0.33**	0.07**	14.6**
H9	0.23*	0.08*	17.02*	0.00^ns^	0.00^ns^	0.21^ns^	0.32**	0.09**	20.7**
H10	−0.09^ns^	−0.08**	−18.3**	−0.02**	−0.0**	−1.5**	0.22**	0.02^ns^	3.60^ns^
H11	−0.07^ns^	−0.05^ns^	−10.1^ns^	−0.01*	0.00**	−1.1**	0.13*	0.01^ns^	3.49^ns^
H12	0.21^ns^	0.09**	19.21**	0.01*	0.00^ns^	0.70*	0.09^ns^	0.04*	8.21*
H13	−0.04^ns^	−0.02^ns^	−5.40^ns^	−0.01*	0.00^ns^	−0.6*	0.17**	0.04*	7.97*
H14	0.11^ns^	−0.01^ns^	−3.09^ns^	−0.01**	−0.0**	−1.2**	0.24**	0.04*	8.43*
H15	−0.25*	−0.07*	−15.2*	0.00^ns^	0.00^ns^	0.12^ns^	−0.07^ns^	−0.02^ns^	−3.9^ns^
H16	−0.08^ns^	0.08*	17.88*	0.03**	0.02**	3.65**	0.11^ns^	0.10**	22.1**
H17	0.25*	0.07*	15.60*	0.00^ns^	0.00^ns^	−0.0^ns^	0.45**	0.12**	27.3**
H18	0.15^ns^	0.18**	38.53**	0.03**	0.02**	4.13**	0.28**	0.17**	37.6**
H19	0.41**	0.14**	31.22**	0.01**	0.00^ns^	0.77*	0.45**	0.14**	30.2**
H20	0.59**	0.07*	16.36*	−0.02**	−0.01**	−2.3**	0.58**	0.10**	21.3**
H21	−0.22*	−0.04^ns^	−7.64^ns^	0.01**	0.01**	0.79*	0.01^ns^	0.02^ns^	3.36^ns^

ns: non-significant, **, *significant at *P*≤0.01 and *P*≤0.05, respectively. AGP: AG percentage per plant (%), AGC: AG content per plant (g), AGY: AG yield per hectare (kg/ha), NAGP: NAG percentage per plant (%), NAGC: NAG content per plant (g), NAGY: NAG yield per hectare (kg/ha), DDAGP: DDAG percentage per plant (%), DDAGC: DDAG content per plant (g), DDAGY: DDAG yield per hectare (kg/ha). P1–7 refers to the parental plants and H1–H21 refers to 21 hybrid plants.

### Specific Combining Ability (SCA)

The phytochemical traits demonstrated different features of SCA estimates, in which both positive and negative significant values existed within the 21 hybrids (Table 5). This situation implied a complex genetic mechanism controlling the phytochemicals in AP. The SCA results revealed a significant variation among the 21 hybrids in which the P1×P7 combination produced the best hybrid (H6) with the highest SCA effects for AG and DDAG contents (0.2** and 0.18**, respectively) (Table 5). Positive and significant SCA effects were shown by hybrids H18 (P4×P7) and H19 (P5×P6) for AGC (0.18**, 0.14**, respectively) and for DDAGC (0.17**, 0.14**, respectively), as well (Table 5). In the case of NAGC, the hybrids H1 (P1×P2), H6 (P1×P7) and H18 (P4×P7) were the most successful crosses with the highest SCA effects (0.03**, 0.02** and 0.02**, respectively). P1 acted as the best maternal parent (♀) for AGC and DDAGC in combination of P1×P7 as well as for NAGC in combination of P1×P2, simultaneously. On the other hand, P7 was a good paternal parent (♂) in the combination of P1×P7 for AGC and DDAGC. Meanwhile, P2 parent performed well as a donor for NAGC in the combination of P1×P2.

### Estimation of Broad and Narrow-sense Heritabilities of the Phytochemicals

Heritability is an important statistical outcome of diallel studies. The broad- and narrow-sense heritability estimates of the three phytochemicals were measured in the hybrids. Highly heritable patterns were observed in neoandrographolide percentage (NAGP), NAGC, 14-deoxy-11,12-didehydroandrographolide percentage (DDAGP) and DDAGC in the broad-sense with values of 81.7, 80.4, 84.1 and 83.3% respectively, while AGP and AGC were determined as moderately heritable traits in the broad-sense with a magnitude of 36.6 and 47.7%, respectively (Table 6). On the contrary, the negative values of GCA variances of the AG and DDAG components, led to negative narrow-sense heritability estimates for both these traits. Nevertheless, slightly different results with low but positive values of the narrow-sense heritability (15.3 and 9%) emerged for NAGP and NAGC, correspondingly (Table 6).

**Table 6:** Estimates of the genetic parameters of the three phytochemicals in a 7×7 half diallel.

Traits	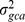	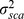	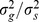		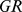		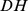	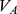	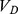	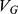	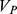	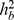	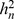
AGP	−0.002793^ns^	0.089067**	−0.03	−0.07		−3.99		−0.005586	0.089067	0.083481	0.228151	36.6	−2.4
AGC	−0.001392^ns^	0.015416**	−0.09	−0.22		−2.35		−0.002784	0.015416	0.012632	0.026449	47.7	−10.5
NAGP	0.000035792^ns^	0.000308839**	0.12	0.19		2.10		0.000071584	0.000308839	0.000380423	0.000465433	81.7	15.3
NAGC	0.000008671^ns^	0.000137444**	0.06	0.11		2.82		0.000017342	0.000137444	0.000154786	0.000192366	80.4	9.0
DDAGP	−0.02516^ns^	0.25356**	−0.1^†^	−0.25		−2.24		−0.05032	0.25356	0.20324	0.24166	84.1	−20.8
DDAGC	−0.002389^ns^	0.024951**	−0.1^†^	−0.24		−2.29		−0.004778	0.024951	0.020173	0.024209	83.3	−19.7

ns: non-significant, **: significant at *P*≤0.01 level, 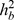: broad-sense heritability (%), 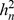: narrow-sense heritability (%), *GR*: genetic ratio, PH: plant height (cm), AGP: AG percentage per plant (%), AGC: AG content per plant (g), AGY: AG yield per hectare (kg/ha), NAGP: NAG percentage per plant (%), NAGC: NAG content per plant (g), NAGY: NAG yield per hectare (kg/ha), DDAGP: DDAG percentage per plant (%), DDAGC: DDAG content per plant (g), DDAGY: DDAG yield per hectare (kg/ha). AGY, NAGY and DDAGY possessed similar heritability values with AGC, NAGC and DDAGC, respectively. Hence, these components are not presented in this table.

### Additive and Dominance Variances

The dominance variances of the andrographolides were higher than their additive variances indicating the prevalence of non-additive effects over the additive gene actions in controlling these phytochemicals (Table 6). According to the definitions of additive and non-additive genetic variations presented by the American Society of Foresters, this model implied the converse of the effects of alleles combining in a linear, incremental fashion to produce genetic variation. In other words, the proportion of genetic variation, which caused specific pairwise crosses to depart from the performance values predicted by the breeding values of the parents, was very notable for the investigated compounds.

### Gene Action and Degree of Dominance

Thus far, three methods of estimating dominance have been applied to the F_1_ data on the AG, NAG as well as DDAG components of the 21 hybrids resulting from diallel crosses among the seven parental AP accessions. However, as a general clue, the preponderance of SCA variances to GCA variances in the AG, NAG, DDAG phytochemicals and their components suggested that the non-additive gene effects were more important than the additive effects in controlling these characteristics (Table 6). In addition, the low ratio of GCA to SCA variances attested the higher proportion of the non-additive gene effects rather than the additive ones for all the three investigated phytochemicals [39], where the values of the aforementioned ratio were found far from unity regardless of their positivity or negativity (Table 6). The data from genetic ratios (*GR*) agreed with the GCA/SCA ratios of AG and DDAG, where the *GR* ratios showed negative values due to the negativity of the numerator (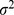
_gca_), means that non-additive effects governed the heritability of AG and DDAG. NAG was inherited under the control of additive effect having *GR* values greater than unity (Table 6). Unlike the *GR* results, the rates of *DH*s verified the GCA/SCA ratios, whereas the existence of non-additive effects (overdominance) was proposed for the control of AG, NAG and DDAG owing to the observation of negative (for AG and DDAG, *DH*<0) and higher than unity values (*DH*>1, for NAG) of *DH* (Table 6).

Finally, the heterosis-based evaluation proved its importance to provide an accurate and more realistic estimate of the degree of dominance for each cross combination compared to the previous assays (Table 7). It was realized that the majority of the phytochemicals and their components in AP were exposed to non-additive (more specific to the overdominance) genetic effects due to the recorded values of *h* (*h*>1). The H2 (*h* = 0.59) and H15 (*h* = –1.54) hybrids were the two exceptional cases exhibiting respectively the partial dominance and negative overdominance effects for AGP, whilst the rest of the hybrids fitted to the positive overdominance model. However, the presence of partial dominance (in H5, H8, H11 and H13), and negative overdominance (in H10, H14 and H20) were detected for NAG. The result of the degree of dominance for DDAG was in accordance with the GCA/SCA and *DH* ratios, as every one of the 21 hybrids was influenced by the overdominance effects (Table 7).

**Table 7:** Heterosis-based degree of dominance of the three phytochemical components in the 21 AP hybrids.

Hybrids	*h*								
	AGP	AGC	AGY	NAGP	NAGC	NAGY	DDAGP	DDAGC	DDAGY
H1	2.31	22.43	22.43	6.11	9.95	9.95	5.48	14.55	14.55
H2	0.59	2.90	2.90	32.08	60.43	60.43	8.89	12.11	12.11
H3	32.07	10.89	10.89	21.35	5.67	5.67	377.86	75.59	75.59
H4	3.42	7.80	7.80	2.43	4.58	4.58	40.21	67.09	67.09
H5	2.14	3.17	3.17	0.97	2.60	2.60	16.92	18.55	18.55
H6	2.40	5.83	5.83	14.17	51.70	51.70	8.27	12.84	12.84
H7	9.41	4.54	4.54	1.17	1.41	1.41	159.75	39.36	39.36
H8	7.28	59.86	59.86	0.18	0.35	0.35	12.13	21.92	21.92
H9	34.57	27.20	27.20	1.75	2.65	2.65	16.68	24.29	24.29
H10	2.35	0.39	0.39	−3.60	−2.91	−2.91	6.30	6.01	6.01
H11	2.25	1.87	1.87	0.89	1.08	1.08	24.23	13.63	13.63
H12	3.10	5.43	5.43	11.05	14.19	14.19	9.18	12.04	12.04
H13	3.66	2.41	2.41	−0.22	0.25	0.25	14.85	11.78	11.78
H14	7.13	53.91	53.91	−2.66	−2.34	−2.34	6.33	5.28	5.28
H15	−1.54	7.98	7.98	6.56	8.37	8.37	13.54	20.98	20.98
H16	4.84	63.56	63.56	6.32	7.22	7.22	46.47	824.64	824.64
H17	3.06	5.17	5.17	0.66	1.01	1.01	18.08	16.60	16.60
H18	2.49	7.57	7.57	12.31	16.57	16.57	8.41	14.30	14.30
H19	6.49	6.88	6.88	8.49	15.04	15.04	14.86	16.40	16.40
H20	7.97	5.36	5.36	−3.48	−2.28	−2.28	14.01	11.73	11.73
H21	10.27	12.43	12.43	5.10	5.79	5.79	4.16	4.66	4.66

*h*: degree of dominance, AGP: AG percentage per plant (%), AGC: AG content per plant (g), AGY: AG yield per hectare (kg/ha), NAGP: NAG percentage per plant (%), NAGC: NAG content per plant (g), NAGY: NAG yield per hectare (kg/ha), DDAGP: DDAG percentage per plant (%), DDAGC: DDAG content per plant (g), DDAGY: DDAG yield per hectare (kg/ha).

### Heterotic Behavior of the AP Hybrids

As a promising result and typically positive breeding response to intraspecific hybridization, a range of positive heteroses in mid- and better-parent levels occurred in most of the hybrids for AG, NAG and DDAG and their components. Even so, the occasional negative heteroses were happened for NAG and its components (Table 8).

**Table 8:** Mid- and better-parent heterosis of the three phytochemicals in the 21 AP hybrids.

Hybrids	Mid-parent heterosis (%)						Better-parent heterosis (%)					
	AGP	AGC†	NAGP	NAGC†	DDAGP	DDAGC†	AGP	AGC†	NAGP	NAGC†	DDAGP	DDAGC†
H1	20.29	88.07	47.03	126.68	112.21	233.31	10.59	80.96	36.52	101.07	76.14	187.25
H2	7.37	43.32	27.12	69.75	171.55	262.58	−4.52	24.69	26.05	67.81	127.64	197.96
H3	37.53	54.18	5.90	18.35	270.21	315.67	35.94	46.87	5.61	14.64	267.58	299.01
H4	25.70	47.54	9.82	28.09	249.26	310.18	16.91	39.06	5.55	20.69	228.87	292.06
H5	32.64	46.74	4.19	14.47	422.72	476.45	15.12	27.89	−0.12	8.44	318.22	358.64
H6	35.57	93.14	34.68	91.14	214.95	347.74	18.04	66.52	31.46	87.83	150.00	252.33
H7	34.98	50.35	8.02	19.61	196.76	230.46	30.14	35.35	1.10	5.04	193.15	212.18
H8	55.39	63.07	1.45	5.54	239.99	261.70	44.41	61.37	−6.04	−8.94	183.82	223.13
H9	43.68	59.25	6.41	17.63	241.22	279.24	41.89	55.85	2.66	10.28	198.11	240.14
H10	15.33	4.20	−12.18	−21.03	272.27	240.85	8.26	−6.02	−15.06	−26.35	159.88	143.34
H11	13.88	22.70	4.68	11.89	140.85	157.34	7.28	9.42	−0.55	0.80	127.62	130.70
H12	35.04	54.52	12.39	29.57	170.72	212.70	21.33	40.42	11.15	26.93	128.26	165.74
H13	18.23	21.48	−0.71	1.81	196.74	203.04	12.62	11.53	−3.80	−5.10	162.02	158.49
H14	20.12	11.19	−9.24	−15.72	267.22	236.78	16.83	10.96	−12.29	−21.02	158.17	132.46
H15	−3.76	8.48	10.52	24.42	95.34	120.18	−6.05	7.34	8.77	20.90	82.49	108.25
H16	30.72	71.47	27.35	67.51	254.94	369.21	22.91	69.56	22.07	53.20	236.48	367.11
H17	43.12	50.87	3.03	8.86	463.76	490.45	25.47	37.36	−1.49	0.07	348.66	355.78
H18	34.05	83.97	33.51	82.81	212.88	331.17	17.89	65.60	29.97	74.11	149.69	250.07
H19	50.59	59.97	2.29	8.46	456.24	491.33	39.71	47.14	2.01	7.86	325.56	355.03
H20	59.05	53.54	−6.53	−9.95	281.65	266.78	48.07	39.61	−8.25	−13.72	217.77	198.82
H21	3.89	15.79	9.56	22.05	198.89	230.04	3.50	14.33	7.54	17.58	102.15	121.01
Average	29.21	47.80	10.44	28.66	244.30	288.91	20.30	37.42	6.80	21.05	187.73	226.17

AGP: AG percentage per plant (%), AGC: AG content per plant (g), AGY: AG yield per hectare (kg/ha), NAGP: NAG percentage per plant (%), NAGC: NAG content per plant (g), NAGY: NAG yield per hectare (kg/ha), DDAGP: DDAG percentage per plant (%), DDAGC: DDAG content per plant (g), DDAGY: DDAG yield per hectare (kg/ha).

† AGY, NAGY and DDAGY possessed the similar heteroses with AGC, NAGC and DDAGC, respectively.

The maximum MPH was observed in hybrids H20 for AGP (59.05%), H6 for AGC (93.14%), H1 for NAGP and NAGC (47.03 and 126.68%), H17 for DDAGP (463.76%), and H19 for DDAGC (491.33%). Most of the negative and lowest heteroses were recorded for NAG and its components in both mid- and better-parent levels, simultaneously. In contrast, not only did DDAG and its components have no negative values, but also they were strongly subjected to the heterosis phenomenon to the extent that hybrids H17 and H19 became the record-breaking cases in DDAGC with 490.45 and 491.33%, respectively (Table 8). Moreover, the results showed that AG was posited in the mid-range of heterosis with the averages of 29.21 and 47.80% in AGP and AGC at the mid-parent level followed by 20.30 and 37.42% of the same components at the better-parent level (Table 8).

### Correlations of the Andrographolides before and after Hybridization

**Figure 4:**
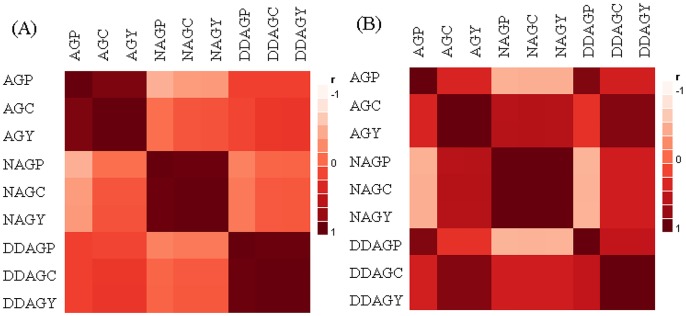
Color maps based on the correlations of the andrographolides in (A) the seven parental plants, and (B) in the 21 hybrids. The strength and trend of the correlations among the studied phytochemicals and their components in this investigation are indicated by the colors which ranged from dark-red to white. Dark-red squares comply with the positive correlations, pink colors refer to the negative correlations, and the light reds represent no correlation status. Shadings are depended to the strength of the correlations. AGP: AG percentage per plant (%), AGC: AG content per plant (g), AGY: AG yield per hectare (kg/ha), NAGP: NAG percentage per plant (%), NAGC: NAG content per plant (g), NAGY: NAG yield per hectare (kg/ha), DDAGP: DDAG percentage per plant (%), DDAGC: DDAG content per plant (g), DDAGY: DDAG yield per hectare (kg/ha).

One of the most remarkable results of this exploration was the documentation of the correlations of the three andrographolides and their components. The correlation analysis unveiled how the relationships of these phytochemicals can be diversified after running intraspecific hybridization (Figs. 4A and 4B). The negative correlations of DDAGP with NAG and its components were highlighted in a significant way (*P*≤0.05) amongst the hybrids (Table 9), while they were not significantly correlated together in the parental APs (Table 10). The negative relationships of AGP with NAG and its components were boosted among the hybrids as it reached a significant level (*P*≤0.05) between AGP and NAGP (Table 9). Surprisingly, the non-significant mode between AGC and NAGP-C in the parental plants was changed after hybridization as they were correlated to each other with significant positive values (Tables 9 and 10). Intriguingly, DDAGC repeated the same trend by showing a significant positive correlation with NAGP and NAGC.

**Table 9:** Correlations of the three phytochemicals in the 21 AP hybrids.

	AGP	AGC	AGY	NAGP	NAGC	NAGY	DDAGP	DDAGC	DDAGY
AGP	1								
AGC	0.42	1							
AGY	0.42	1**	1						
NAGP	−0.44*	0.63**	0.63**	1					
NAGC	−0.42	0.64**	0.64**	0.99**	1				
NAGY	−0.42	0.64**	0.64**	0.99**	1**	1			
DDAGP	0.90**	0.32	0.32	−0.47*	−0.45*	−0.45*	1		
DDAGC	0.45*	0.88**	0.88**	0.47*	0.47*	0.47*	0.55**	1	
DDAGY	0.45*	0.88**	0.88**	0.47*	0.47*	0.47*	0.55**	1**	1

**, *Correlation is significant at the 0.01 and 0.05 levels, respectively (2-tailed). AGP: AG percentage per plant (%), AGC: AG content per plant (g), AGY: AG yield per hectare (kg/ha), NAGP: NAG percentage per plant (%), NAGC: NAG content per plant (g), NAGY: NAG yield per hectare (kg/ha), DDAGP: DDAG percentage per plant (%), DDAGC: DDAG content per plant (g), DDAGY: DDAG yield per hectare (kg/ha).

**Table 10:** Correlations of the three phytochemicals in the seven parental AP.

	AGP	AGC	AGY	NAGP	NAGC	NAGY	DDAGP	DDAGC	DDAGY
AGP	1								
AGC	0.91**	1							
AGY	0.91**	1**	1						
NAGP	−0.43	−0.03	−0.03	1					
NAGC	−0.31	0.11	0.11	0.97**	1				
NAGY	−0.31	0.11	0.11	0.97**	1**	1			
DDAGP	0.22	0.19	0.19	−0.14	−0.09	−0.09	1		
DDAGC	0.22	0.27	0.27	0.02	0.09	0.09	0.98**	1	
DDAGY	0.22	0.27	0.27	0.02	0.09	0.09	0.98**	1**	1

**, *Correlation is significant at the 0.01 and 0.05 levels, respectively (2-tailed). AGP: AG percentage per plant (%), AGC: AG content per plant (g), AGY: AG yield per hectare (kg/ha), NAGP: NAG percentage per plant (%), NAGC: NAG content per plant (g), NAGY: NAG yield per hectare (kg/ha), DDAGP: DDAG percentage per plant (%), DDAGC: DDAG content per plant (g), DDAGY: DDAG yield per hectare (kg/ha).

## Discussion

Determination, variation and stability of the andrographolides in AP are not novel topics, while they have been investigated previously [29], [48], [49], but unfortunately, the genetic aspects as well as the precise heritability features of these phytochemicals are still uncovered. To this end, the diallel-based researches to gauge the feasibility of the genetic enhancement of the key andrographolides of AP are proposed. Undoubtedly, the heterosis of AG is an exception since its occurrence has very recently been revealed as a part of this investigation [27]. However, from this point of view, the current experiment deserves a “first report”. At a glance, the content of AG was higher than DDAG and NAG in an order of AG>DDAG>NAG. Interestingly, this was in agreement with the outcomes of the previous trials [49], [50].

The highest rate of heterosis was recorded for DDAGC with the averages of 288.91% and 226.17% in the mid- and better-parent levels, respectively, by following an order as DDAG>AG>NAG. However, the high magnitude of heterosis for DDAG did not disarrange the order of the total andrographolides contents (AG>DDAG>NAG). According to the overdominance hypothesis in genetics, the certain combinations of alleles which can only be obtained by outbreeding are especially advantageous for the existence of hybrid vigor or heterosis when paired in a heterozygous individual [42]. High values of heterosis in a certain trait are the result of non-additive genes and are especially linked to the overdominance effects [44], [51]–[53].

A theoretical interpretation of the obtained results (the preponderance of non-additive gene actions) is that the interactions of the genes involved in the biosynthesis of the three andrographolides of AP, are likely to generate interaction at the level of the variance for these phytochemicals. This is opposed to the situation that Hill et al. [68] had explained about complex traits. In spite of the allelic interaction, the incidence of heterosis has been classically referred to as the overdominance model [58]. The impact of other gene actions such as epistasis should not be entirely ruled out for complex traits particularly in self-pollinated crop species [59]. The non-additive type of gene action is desirable for heterosis breeding and might be exploited in hybrid seed production, while the additive type of gene action is suitable for the simple selection method [43]. For this reason, producing hybrid seeds for AP is more rational than improvements through the simple selection method due to the lack or imperceptible proportion of additive gene action in these traits. According to Williams et al. [36], the partial dominance hypothesis attributes the inbreeding depression to increased homozygosity of alleles which are both deleterious and partially recessive. The overdominance hypothesis is based on the higher fitness of a heterozygote over either homozygote or inbreeding depression arises from a loss of heterozygosity [36]. This exactly fits the situation that Malaysian AP has been encountered with. On the one hand, a high level of homozygosity along with a special type of monomorphic heterozygosity (fixed heterozygosity) was revealed using microsatellite markers [54]. Further, randomly amplified polymorphic DNA (RAPD) markers indicated a low genetic diversity among the Malaysian AP populations [27].

Essentially, the evolutionary dynamics of the AP plant was not concerned as one of the main objectives of the present study. However, taking these aspects into consideration help us to achieve a better understanding of the genetic basis of the anticancer andrographolides in AP. Generally, the overdominance genetic action of the analyzed andrographolides in AP could probably be attributed to the self-pollinated mating system of this plant and a consequent inbreeding depression [23]. As a matter of fact, inbreeding depression in self-pollinated plant species has received a little attention [59]. The presence of a subtle level of this phenomenon in AP has been noticed recently [27], and we assume that a part of the detected heterosis could be generated because of suppressing the genetic depression in the F_1_ hybrids. However, this behavior could have specifically been intensified in the bottlenecked population of AP in Malaysia [54]. In light of the convincing molecular evidences on the Malaysian AP populations [27], [54], the use of an F_2_ population was dispensable to detect outbreeding depression. Evidently, F_2_ plants are employed when outbreeding depression might not be perceived in the F_1_ generation due to high heterosis, and might only appear in the next generations. However, this is prevalent in self-compatible plants that their flowers are naturally considered to be predominantly outcrossed [69], while AP is far from this situation.

The relative proportion of additive and non-additive variation for quantitative traits is important in evolutionary biology, medicine, and agriculture [68]. According to the neutral quantitative genetic theory, population bottlenecks are expected to decrease the standing level of additive genetic variance (*V_A_*) in quantitative traits [55]–[57]. Smaller amounts of additive variances (*V_A_*) shown in table 6 are supporting this concept. Based on Wright’s theory [60], the additive genetic variance within a population (following a bottleneck or inbreeding) is anticipated to decrease the inbreeding coefficient of the population. This ensues when genetic variation underlying a quantitative characteristics controlled by genes that “act additively” within and between loci [70]. Hence, an evolutionary perspective could be drawn that these anticancer factors were originally controlled by additive gene action in the Indian ancestors of AP, however, their additive variance decreased because of a bottleneck event after their introduction to Malaysia [54]. The latter assumption gives raise the need for future studies to investigate the role of additive gene action in controlling the heritability of the andrographolides using different AP populations.

Degree of dominance takes an important place in diallel-based studies, and different methods may lead to various results by using the same data. Therefore, this point should be emphasized strictly, because the breeding endeavors may mislead seriously upon an inaccurate estimation of the gene action.

**Figure 5:**
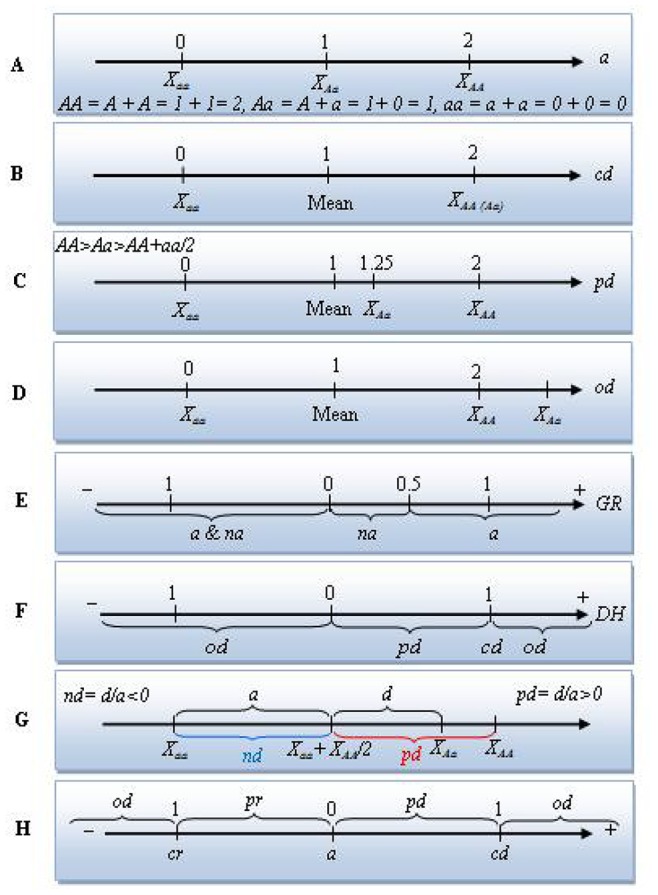
Schematic illustration of the classic definition of different gene actions for a biallelic, locus “X” with homozygote genotypes “aa” (the recessive alleles) and “AA” (the dominant alleles), and heterozygote genotype “Aa” (hybrid). A: additive gene action, B: complete dominance gene action that causes the same values for both dominant heterozygote and homozygote individuals, C: partial dominance gene action, D: overdominance gene action, E: gene actions based on the genetic ratio (*GR*) values, F: gene actions based on the *DH* values, G: additive and dominance gene actions described by Gjuvsland et al. [45] (doi:10.1371/journal.pone.0009379.g001), the blue accolade (*nd* area) and the red accolade (*pd* area) have been added to the original version of the image, H: gene actions based on the Petr and Frey (1966), and Falconer (1989) formulas. *a*: additive, *na*: non-additive, *d*: dominance, *nd*: negative dominance, *cd*: complete or full dominance, *cr*: complete of full recessive, *pd*: partial dominance, *pr*: partial recessive, *od*: overdominance.

In line with this, some of the advantages and disadvantages of the applied methods are discussed. Apart from the overlapping of additive and non-additive effects based on the *GR* values, the dominance and over-dominance effects are expressed under one category stated as non-additive. In other words, not only are the *GR* values not able to differentiate between full and partial dominance, but also this index is incapable of differentiating the dominance and over-dominance effects from each other (Fig. 5E).

Although, every three approaches for estimating the degree of dominance confirmed one another, the Petr and Frey’s strategy is more fascinating than the other designs for several reasons. First of all, there is no overlapping between the additive and non-additive gene actions areas as shown in Figure 5H. Secondly, the borders between partial dominance and complete dominance as well as the edges of partial recessive and complete recessive are clearly distinct. Thirdly, the Petr and Frey’s procedure allows to estimate the gene action for each trait and each combination (diallel cross) separately, which is totally unachievable using the other procedures. Fourthly, the negative dominance area does not merge with the additive effects as this may arise in some calculations (Fig. 5G). This situation arises with assuming; *a = X_AA_ – X_aa_/2* and *d = X_Aa_ – (X_aa_+X_AA_)/2*, for classical additive and dominance genotypic values “*a*” and “*d*” of a biallelic locus [47]. Consequently, if the heterozygote has a genotypic value less or greater than both homozygotes (*d/a*<−1 or *d/a*>1), the locus shows negative or positive overdominance, respectively, with the term overdominance covering both cases (|*d/a*|>1). If *d* = 0, the locus shows additive gene action. When *d*/*a* is positive, the heterozygote has a genotypic value larger than the means of the two homozygotes, and the locus demonstrates positive dominance (or positive non-additive gene action). In addition, it is stated that if *d*/*a* is negative, the heterozygote is positioned below the mean value, and the locus exhibits negative dominance (recessive, or negative non-additive gene action) [47]. Obviously, the last part (the negative value of *d*/*a*) causes a great confusion as the additive gene action area is mixed with the negative dominance region. This situation has been highlighted with the blue accolade in Figure 5G.

**Figure 6:**
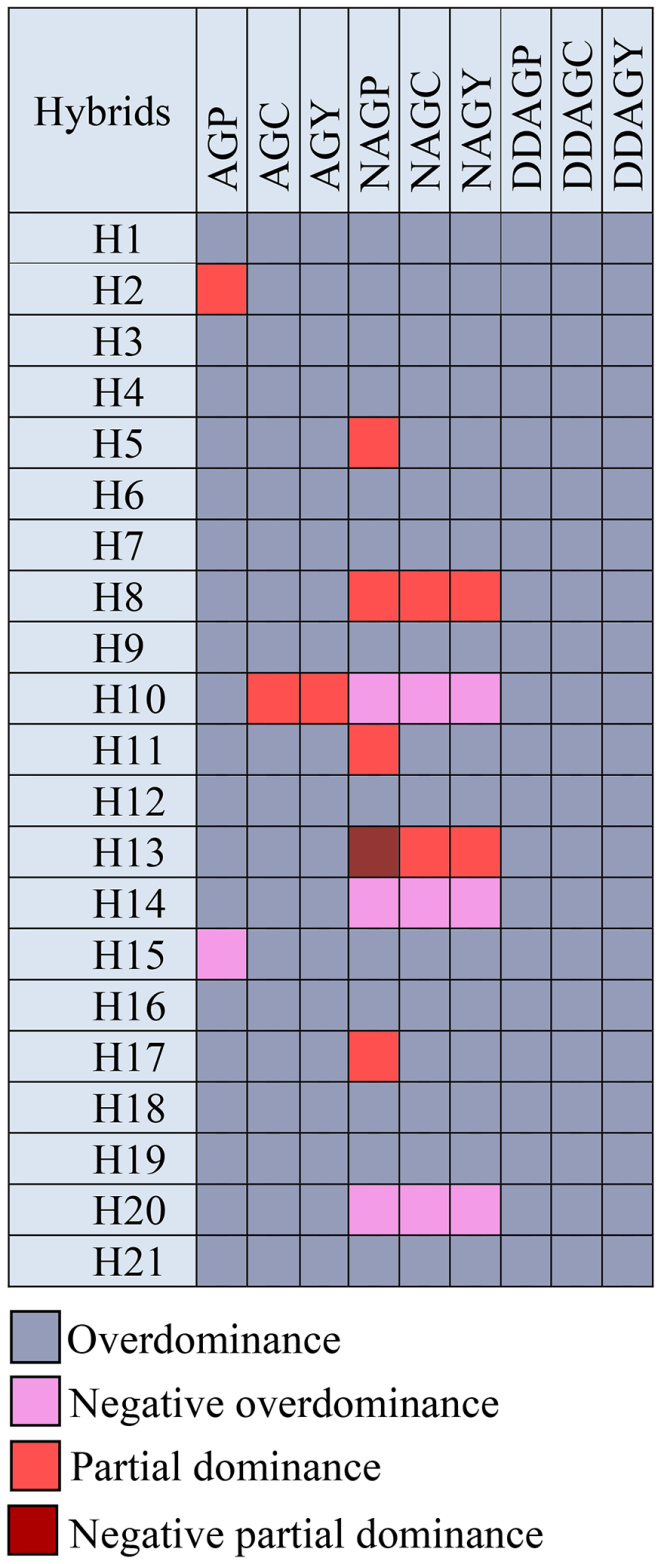
Graphical demonstration of the heterosis-based degree of dominance of the three anticancer andrographolides and their components in AP hybrids. AGP: AG percentage per plant (%), AGC: AG content per plant (g), AGY: AG yield per hectare (kg/ha), NAGP: NAG percentage per plant (%), NAGC: NAG content per plant (g), NAGY: NAG yield per hectare (kg/ha), DDAGP: DDAG percentage per plant (%), DDAGC: DDAG content per plant (g), DDAGY: DDAG yield per hectare (kg/ha). H1–H21 represent the 21 hybrids.

Thus, we conclude that the logic behind the use of multiple approaches to define the main gene action controlling traits is the ambiguity of the outcomes in GCA and SCA-based calculations [37]. In spite of minute deviations, the non-additive or the overdominance gene action was the most recommendable genetic mechanism controlling the heritability of the three andrographolides in AP (Fig. 6).

The correlations of these andrographolides among the 21 hybrids should also be taken into consideration. Alteration of the content of the andrographolides and especially DDAG are a time-dependent event, which may lead to the considerable fluctuations in their content during the storage time [49]. However this issue is addressed as the harvested plant materials were dried immediately and subjected to the extraction process soon after. Subsequently, the samples were injected into the HPLC with no waste of time. The changes in the correlation of the andrographolides and their components could be attributed to genetic factors driven by outcrossing. These changes are incredibly favorable when most of the modern clinical tests are being carried out using DDAG and AG [61]–[64]. Fortunately, the contents of both these compounds (DDAG and AG) showed the highest increase due to heterosis. Moreover, the role of NAG in clinical researches should not be underestimated.

The high heritability of AG, NAG and DDAG in the broad-sense has been reported very recently [65]. A similar report has been released about the morphological characteristics involved in salt tolerance in AP [66]. Regardless of the non-diallelic methods, the recorded heritabilities could be interpreted as being compatible outcomes with our present results, suggesting that despite all the difficulties associated with, AP has a high potential to be subjected to the intraspecific hybridization or outcrossing [23], [27].

These outcomes could be regarded as promising information for all those who are engaged with programs focused on the plant-based bioactive molecules as the same approach can be utilized in different types of herbal plants especially in the developing countries.

### Conclusion

Hunger still remains a painful reality for the world’s poor and marginalized people [67]. Although symbolically under international obligation, rice (*Oryza sativa* L.) will be preferred over rice bitters (*Andrographis paniculata*), nevertheless, plant breeders must also try their best to make the medicinal plants as productive as possible to get more yields by utilizing less land. Employing the basic principles of genetics proved the feasibility of enhancing the contents of the bioactive molecules in AP. Due to the detection of the non-additive type of gene action, heterosis breeding is proposed to produce hybrid seeds of AP. The resulting prolific AP hybrids with low ecological demands can be introduced carefully to tropical areas with relatively fertile soils (and even poor soils) as a trustworthy source of versatile anticancer andrographolides for use as novel pharmaceutical compounds.

## Materials and Methods

### Plant Materials, Pollination Scheme, Growth Condition and Field Trial

A total of seven AP accessions representing six states of Peninsular Malaysia were manually outcrossed with each other in all 21 one-way possible combinations using a 7×7 diallel cross design described by Valdiani et al. [23], [27] as shown in Table 1. Ultimately, a sum of 28 samples (10 seeds of each) consisting of seven parental plants and 21 progenies was grown and tested using a field pot trial. The field pot trial was used as the preferred planting design previously described by Valdiani et al. [27]. The seeds were germinated according to Talei’s protocol [26]. Ten-day seedlings were then transferred into the Jiffy media at the two-leaf stage. The second transplantation was conducted over thirty days and 6–8 leaf seedlings were transferred into the polybags [27].

To verify the reliability of the results, field experiments were carried out at two different planting seasons in open area at Technology Garage of Universiti Putra Malaysia based on a Randomized Complete Block Design (RCBD) experimental design with five replicates.

### Plant Extracts Isolation and Sample Preparation

Aerial parts of the plants were harvested before flowering and were dried in a universal ventilated-electric oven (Memmert, Germany) at 55°C for 48 hours. Dried materials were ground into a fine powder and kept in zipped plastic bags at –20°C for a very short period. A 1∶1 (v/v) mixture of DCM and methanol were used for extraction in which materials were soaked for three days at room temperature. The process was repeated several times with the same solvent system until the solvent turned colorless. The solvent extracts were then filtered using Whatman No. 1 filter paper. The filtered extracts were concentrated under reduced pressure using a rotary evaporator and were then transferred into conical flasks and the residual solvent was removed. A final drying procedure was performed by placing the concentrated extract in the same electric oven adjusted to room temperature. The well-dried extracts were placed into small glass containers, sealed and stored at –20°C. For High performance liquid chromatography (HPLC) analysis, 1 mg of each sample was dissolved in 1 mL of HPLC grade methanol (Merck, Germany) out of which, 20 µL was filtered into HPLC vials using disposable polypropylene syringe filters (pore size of 0.2 µm) just prior to analysis.

### Standards, Solvents and Equipments

AG (98%) was supplied by Sigma-Aldrich, USA. The other two phytochemicals (DDAG and NAG) were obtained from in-house standards collection. Solvents (AR grade) used for isolation and purification of the compounds were supplied by Fisher Scientific (UK). Silica gel (70–230 MESH) and 20×20 cm silica gel 60 F254-coated TLC plates were purchased from Merck (Darmstadt, Germany). In addition, HPLC grade solvents including methanol and acetonitrile were provided by Merck (Darmstadt, Germany). The HPLC system was supported by Waters™ and consisted of Waters™ 600 Controller pumps, Waters™ 717plus Autosampler injector with a capacity of 96 samples. LiChrocart® HPLC-Column RP-18 (150×4.6 mm, Merck, Germany) was used as the stationary phase. The isocratic mobile phase was implemented with acetonitrile- water (40∶60 v/v) and 0.1% (v/v) analytical grade phosphoric acid dissolved in ultra-pure water at a flow rate of 1 mL/min [28]. The water used in this research was purified using the MilliporeTM water purification system. Detection was done at 223 nm using Waters™ 486 Tunable Absorbance Detector (photodiode array detector).

### HPLC Analysis, Calibration Curves of Standard Samples

The stock solutions of the standard samples of AG, NAG and DDAG were prepared at 1 mg/mL concentration using HPLC grade methanol. The stock solutions were then diluted with the same solvent to obtain concentrations ranging from 0.1 to 1000 µg/mL. Consequently, 20 µL of each dose of the working standard solutions was injected in five replicates into the HPLC apparatus. A calibration curve was generated by linear regression based on peak areas [28]. To check the sensitivity of the method, the limit of detection (LOD) was calculated on the basis of a signal-to-noise ratio (S/N) of 3 and the limit of quantification (LOQ) was calculated as 10 times the baseline noise level [29].

### Chemical Structure Display

The 2D and 3D structures of the three phytochemicals were drawn using MarvinSketch 5.11.1 program (Fig. 1A–F).

### Diallel Analysis

The diallel analysis was conducted following Griffing’s Model 2 (random effect) and Method 2 (parents+F_1_ progenies), while no specific assortment was considered for the parental plants [30]. The data were analyzed using a linear model described by Zhang et al. [31] as follow:
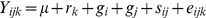
(1)


Where:

Y_ijk_: observed value of each experimental unit,

Μ: mean of the population,

r_k_: replication effects,

g_i_: GCA effects of the i^th^ parent,

g_j:_ GCA effects of the j^th^ parent,

s_ij:_ SCA effects for ij^th^ F_1_ hybrid, and

e_ijk_: residual effect

### General Combining Ability (GCA)

The GCA is defined as the average performance of a particular inbred in a series of hybrid combinations [34]. According to the American Society of Foresters, another definition for GCA is the relative ability of an individual to transmit the genetic superiority to its offspring when crossed with other individuals. The variance of GCA could be estimated using the equation below [31]:
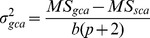
(2)


Where:


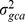
: the variance of GCA

*MS_gca_*: mean square of general combining ability,

*MS_sca_*: mean square of specific combining ability,

*b*: number of replications, and

*p*: number of parents

### Specific Combining Ability (SCA)

The SCA is a performance of a particular parent, in a specific cross. In other word, the SCA is a component of genetic variance calculable where a number of genotypes are intercrossed in all possible combinations. The SCA measures the deviation of the performance of a particular cross from the average general combining ability of its two parents [34]. The variance of SCA can be calculated as below [31]:
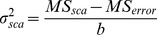
(3)


Where:


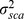
: the variance of SCA

*MS_sca_*: mean square of specific combining ability,

*MS_e_*: mean square of error,

*b*: number of replications, and

*p*: number of parents

### Estimation of *V_A_, V_D_, V_G_* and *V_P_*

The additive gene variation (*V_A_*) is the proportion of genetic variation due to the effects of additive genes (Fig. 5A) that responds to natural selection, mass selection, or pick-the-winner selection. The additive gene variation is the basis of a parent’s breeding value or GCA (Eqns. 4 and 5).
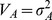
(4)




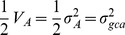
(5)


The combination of equations 4 and 5 results in the following equation:
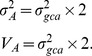
(6)


The dominance gene variation (*V_D_*) is the component of non-additive genetic variation due to within-locus dominance deviations (Fig. 5B). The dominance genetic variation is often used as shorthand for the portion of non-additive genetic variation estimated by full-sib/half-sib mating designs as below:
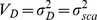
(7)


The genotypic or genetic variance *V_G_* is a sum of the additive and dominance variances (Eq. 8).
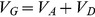
(8)


However, by taking the equations 6 and 7 into consideration, the genetic variance could be obtained using equation 9.
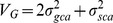
(9)


Where:


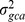
: the variance of GCA, and


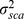
: the variance of SCA

Another way to calculate the *V_G_* is as follows:
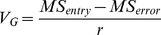
(10)


Where:

MS _entry_: mean square of entry or genotype

MS _error_: mean square of error, and

r: number of replicates

Theoretically, the phenotypic variance (*V_P_*) is the sum of the genetic variance (*V_G_*) and environmental variance (*V_E_*) as shown in equation 11.
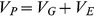
(11)


Considering the equation 9, *V_P_* could be expressed as follows:
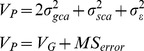
(12)


### Heritability

Broad-sense 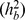 and narrow-sense 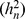 heritability values were calculated based on the variance components [32] in the ANOVA table using the following equations (Eqns. 13, 14,15 and 16).
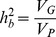
(13)


According to the equations 9, 11 and 12:
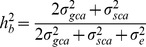
(14)




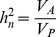
(15)


According to the equations 6 and 12:
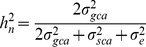
(16)


Where:

*V*_G_ : genetic variance

*V*_A_ : additive variance

*V*_P_ : phenotypic variance


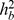
: broad-sense heritability


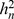
: narrow-sense heritability


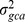
: the variance of GCA,


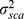
: the variance of SCA, and


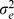
: the variance of error (here the MS of error)

Heritability estimates were classified as low if values were lower than 20%, moderate if the estimates ranged between 20 and 50%, and high if values were larger than 50% [33].

### Gene Actions and Degree of Dominance

The genetic basis (effect) of the three andrographolides was estimated by different approaches in general and specific senses. The average level of dominance was calculated using the genetic ratio (*GR*) suggested by Baker (1978) as shown in equation 17 [35]:
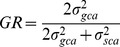
(17)


Where:


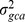
: the variance of GCA


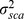
: the variance of SCA

Such that the closer values to unity (*GR* ≈ 1) as well as the values larger than unity (*GR*>1) comply with the greater probability of progeny performance based on GCA (additive) effects, while the values less than 0.5 and closer to zero (0≤ *GR* ≤0.5) agree with the presence of non-additive gene effects. Mathematically, the negative *GR* values can be in accordance with the existence of both additive and non-additive gene actions (Fig. 5E). So that, if the negativity is due to the numerator, this could be explained by non-additive effects, but if the negativity is related to the denominator, this is interpreted with the presence of additive effects.

Furthermore, the relative weight of general and specific combining ability (additive and non-additive gene action) on offspring performance was confirmed at the ratio of GCA variance to SCA variance (σ^2^_gca_/σ^2^_sca_), whereas a value larger than one indicates the additive genetic effect. By contrast, a σ^2^_gca_/σ^2^_sca_ ratio with a value lower than one indicates the non-additive (dominant) genetic effect.

The degree of dominance (*DH*) as shown in equation 18 was used as a confirmatory metric to the *GR* values.
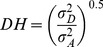
(18)


According to equation 18, if dominance is complete (full) at all loci (*DH* = 1), while, the DH values less than unity (*DH*<1), they collectively indicate the existence of partial dominance. On the other hand, the negative values of *DH* (*DH*<0) as well as the *DH*s larger than unity (*DH*>1) reveal the existence of overdominance for a trait [35].

Regarding the aforementioned deficiencies of the *GR* and *DH* indices, seemingly the level of dominance could be more precisely assessed using the Petr and Frey (1966) formula [37], explained as equation 19.
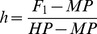
(19)


Where:

*H*: degree of dominance,

*F_1_*: hybrid value,

*MP*: mid-parent value, and

*HP*: high-parent value (better-parent value)

Based on the *h* value, the degree of dominance is classified as: *h* = 0 if there is no dominance, *h* = 1 or *h* = –1 if dominant or recessive is full, 0<*h*<1 if the partial dominance exists, –1<*h*<0 for recessive partial, and *h*>1 or *h*<–1 in case of the presence of overdominance [37].

The Petr and Frey’s equation has in fact been represented again by Falconer (1989) with a little modification in the formula’s components as explicated in equation 20 making it possible to compute the degree of dominance even at the level of a single locus [38].
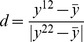
(20)


Where:

*d*: degree of dominance,

*y^12^*: hybrid value,


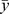
: mid-parent value, and

*y^22^*: high-parent value (better-parent value)

When *d* = 0, the locus is said to show additive gene action (additivity), when 0<|*d*|<1, it shows negative or positive partial dominance, when |*d*| = 1 it shows negative or positive complete dominance, and when |*d*|>1 it is a sign of negative or positive overdominance [39].

### Heterosis

Heterosis is estimated as the percentage of the superiority of the hybrid over its mid-parent value (MP) or better-parent value (BP). The heterosis estimates were presented as equations 21 and 22 for each trait, as follows [40].
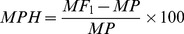
(21)




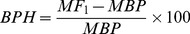
(22)


Where:

*MPH*: mid-parent heterosis,

*BPH*: better-parent heterosis,

*MF_1_*: hybrid value,

*MP*: mid-parent value, and

*MBP*: better-parent value.

### Statistics

The SAS (Statistical Analysis Software) program version 9.1 [45] was used for means comparison analysis of the phytochemicals. Duncan’s multiple range test was performed for means comparison at α = 0.05 and 0.01. We performed the diallel analysis using DIALLEL-SAS05 program [31]. The graphical presentations in Figures 5 and 6 were prepared using Microsoft Word 2010 software. Figures 3 and 4 were prepared using Microsoft Excel 2010 and JMP-8 software [46], respectively. All equations were created using MathType 6.9 software.
